# Heart Failure Risk in Hypertension: Beyond Left Ventricular Hypertrophy

**DOI:** 10.1016/j.jacadv.2026.103317

**Published:** 2026-09-22

**Authors:** Maria Giulia Bellicini

**Affiliations:** Cardiology Institute, Brescia, Italy

Hypertension and left ventricular hypertrophy (LVH) are well-established risk factors for heart failure (HF). However, isolated hypertensive LVH has not been shown to progress by itself to heart failure with preserved ejection fraction (HFpEF) or reduced ejection fraction (HFrEF). Rather, LVH may remain a compensated response to chronic afterload, with additional cardiac abnormalities or insults required for clinical HF to develop. In this context I read with great interest the study by Kaze et al^1^ examining the association between prediabetes, malignant left ventricular (LV) hypertrophy (LVH), and incident heart failure (HF) among hypertensive participants from SPRINT. The authors demonstrate that the combination of electrocardiographic LVH and cardiac biomarker elevation identifies individuals at substantially increased risk of subsequent HF. However, these findings also highlight an important distinction between cardiovascular risk stratification and the identification of a causal maladaptive myocardial phenotype.

In the study, malignant LVH was defined as electrocardiographic LVH accompanied by either high-sensitivity cardiac troponin I ≥ 6 ng/L in men or ≥ 4 ng/L in women, or N-terminal pro–B-type natriuretic peptide (NT-proBNP) ≥125 pg/mL. These thresholds undoubtedly carry prognostic information, but their biological interpretation deserves caution because these biomarkers are strongly influenced by factors other than hypertensive myocardial disease. The high-sensitivity cardiac troponin I concentrations used to define “subclinical myocardial injury” are substantially below the sex-specific 99th percentile upper reference limits reported for the assay.^2^ Furthermore, few hundred elevation in high-sensitivity cardiac troponin is not specific for an intrinsic myocardial disease phenotype and may occur even in structurally normal hearts (even without obstructive coronary artery disease) during transient physiological or hemodynamic stress, including tachycardia. Likewise, NT-proBNP ≥125 pg/mL is an operational threshold used to exclude HF also based on population 99th percentile rather than a biological boundary separating adaptive from maladaptive hypertensive remodeling.^3^ NT-proBNP varies with age, renal function, cardiac rhythm, and cardiac loading conditions.^4^

The lack of cardiac imaging in the analysis by Kaze et al^1^ becomes particularly important in this context. Electrocardiogram voltage criteria cannot distinguish increased LV mass from alternative electrical phenotypes and do not provide direct information on LV geometry, systolic or diastolic function, or other structural cardiac abnormalities. Therefore, electrocardiogram-defined LVH cannot by itself establish the presence of a maladaptive hypertensive myocardial phenotype. These distinctions are particularly relevant when considering the natural history of hypertensive remodeling. More broadly, hypertension and LVH are unequivocally associated with cardiovascular events, but association with future HF should not be equated with demonstration of a causal hypertensive cardiomyopathy. Epidemiological risk, hemodynamic vulnerability, and intrinsic myocardial disease remain distinct concepts.^5^

Consistent with this distinction, in our single-center echocardiographic experience of approximately 2,000 hypertensive outpatients evaluated for hypertensive organ damage, LVH was common whereas left and right ventricle dilatation or dysfunction or hemodynamic features of HF were rare. Conversely, among approximately 770 patients evaluated for acute HF by echocardiography performed at admission before treatment, almost none of those presenting with congestion and preserved or mildly reduced ejection fraction had isolated hypertensive heart disease; instead, an HFpEF mimic was identified, most commonly severe valvular heart disease.^6^ Such observations reinforce the possibility that hypertensive hypertrophic remodeling may frequently represent a compensated response to chronic afterload rather than, by itself, evidence of an intrinsically failing myocardial state. This interpretation does not diminish the well-established adverse prognostic significance of LVH, but distinguishes cardiovascular risk from evidence of causal myocardial failure. Consistently, available longitudinal evidence has not established isolated hypertensive LVH as a condition that predictably progresses toward HFpEF or HFrEF in the absence of superimposed myocardial injury or other structural disease; the concept of a direct transition from hypertensive LVH to HF therefore remains a pathophysiological theory rather than a demonstrated disease trajectory.

I therefore suggest that the phenotype identified by Kaze et al^1^ may be more appropriately regarded as LVH associated with a high cardiovascular-risk biomarker profile, rather than evidence that hypertensive LVH itself has become biologically “malignant.” Prospective studies incorporating cardiac imaging and characterization of subsequent HF phenotypes are needed to clarify this distinction. Biomarkers may identify remarkably well who is at risk, while providing considerably less certainty regarding why that risk exists.
